# Supplementary material for: Effectiveness of a third BNT162b2 mRNA COVID-19 vaccination during pregnancy: a national observational study in Israel
Source: Nat Commun. 2022 Nov 15;13:6961. doi: 10.1038/s41467-022-34605-x (PMC9664047; doi:10.1038/s41467-022-34605-x)
Supplement: Supplementary file 1 — Supplemental Table [file 41467_2022_34605_MOESM1_ESM.pdf]

Supplemental Table 1: COVID-19 related infection and hospitalization according to study period and vaccine status

	Delta period [August 1, 2021-December 1,2022]			Omicron period [December 15, 2021-March 22, 2022]		
	3-Dose	2-dose	Unvaccinated	3-Dose	2-dose	Unvaccinated
N	28303	51942	30627	17123	8612	8282
SARS-CoV-2 positive result	268 (0.9)	2629 (5.1)	2090 (6.8)	3609 (21.1)	1747 (20.3)	1089 (13.1)
COVID-19 Hospitalization:						
All	10 (0.04)	105 (0.20)	341 (1.11)	260 (1.5)	217 (2.5)	207 (2.5)
Significant disease (out of hospitalized cases)	0	4 (3.8)	108 (31.7)	1 (0.4)	5 (2.3)	9 (4.3)
Significant disease (out of total cohort)	0	4 (0.01)	108 (0.35)	1 (0.01)	5 (0.06)	9 (0.11)
Severe disease (out of hospitalized cases)	0	3 (2.9)	64 (18.8)	0	1 (0.5)	5 (2.4)
Severe disease (out of total cohort)	0	3 (0.01)	64 (0.21)	0	1 (0.01)	5 (0.06)
COVID-19 hospitalization duration (days):						
All	2.4 (±1.4)	3.5 (±3.7)	5.2 (±7.8)	4.9 (±6.2)	4.1 (±4.6)	3.9 (±4.4)
Significant disease	-	5.8 (±3.9)	8.6 (±11.8)	-	12.2 (±17.9)	8.8 (±13.4)
Severe disease	-	5.7 (±4.7)	12.1 (±14.2)	-	-	12.8 (±17.7)
Maternal death related to COVID	0	0	1	0	0	0

Data are n (%), and mean (± standard deviation). The study period populations were 82,659 and 33,303 in each of the two periods. In each study period 28,213 and 714 individuals were first included in the 2-dose group and then re-recruited to the 3-dose group. Calculation of COVID related hospitalization is according to follow-up time in each study group until an event occurred, as described in the Methods.

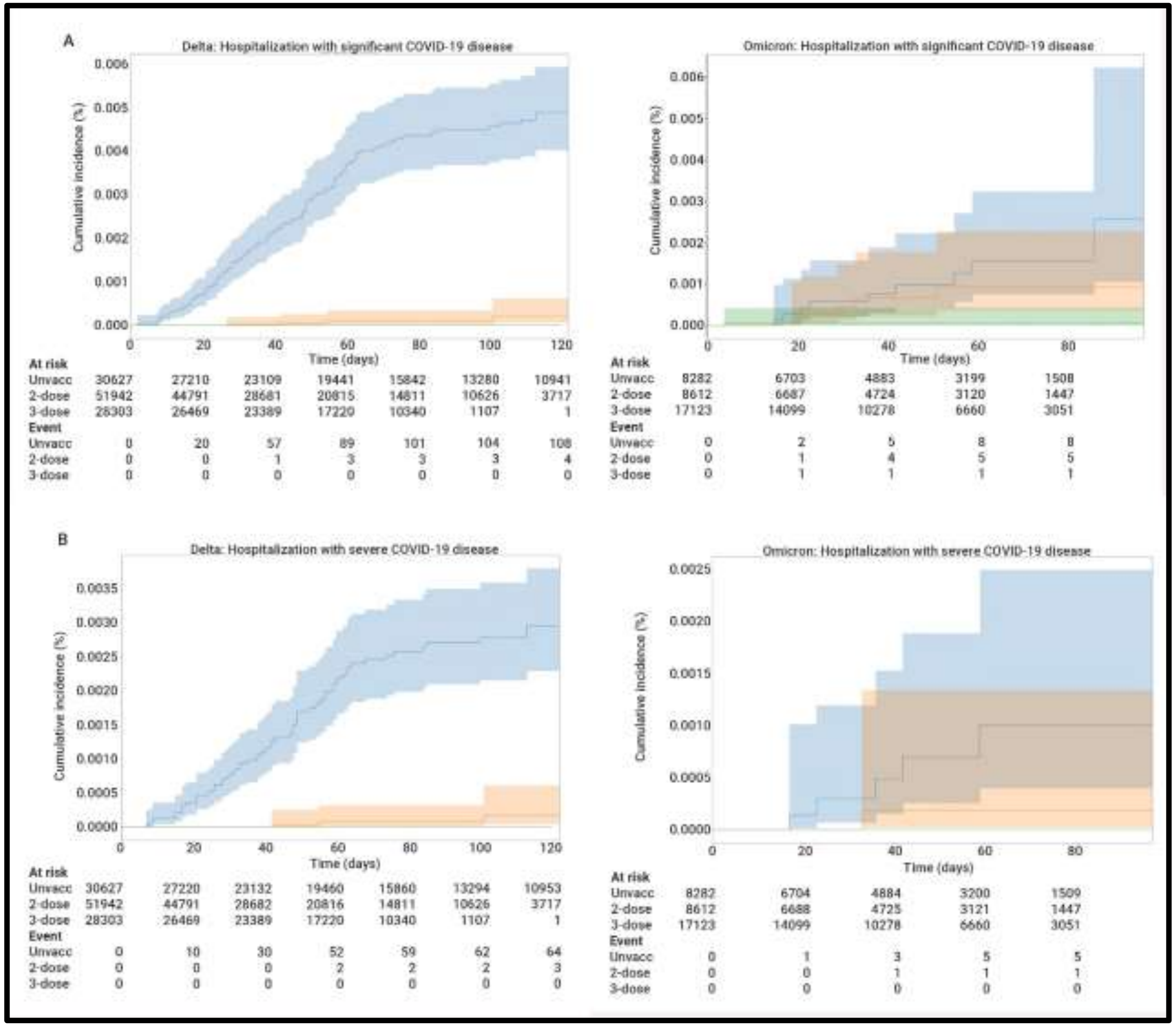

Supplemental Figure 1: Cumulative incidence of study outcomes, according to COVID-19 waves and vaccination status. Cumulative incidence curves comparing the two study periods (Delta period on the left and Omicron period on the right) for hospitalizations with significant disease (A), and severe disease (B) in pregnant women, by vaccination status (Third vaccine-green line, second vaccine-orange line, and unvaccinated- blue line). The main line is the point estimate of the cumulative incidence and shaded areas represent 95% confidence intervals. The number at risk at each time point and the cumulative number of events are also shown for each outcome.
